# Identification of Diagnostic Biomarkers and Their Correlation with Immune Infiltration in Age-Related Macular Degeneration

**DOI:** 10.3390/diagnostics11061079

**Published:** 2021-06-12

**Authors:** Yuyang Zeng, Xiujuan Yin, Changzheng Chen, Yiqiao Xing

**Affiliations:** 1 Eye Center, Renmin Hospital of Wuhan University, 238 Jiefang Road, Wuhan 430060, China; yuyangz@whu.edu.cn; 2 State Key Laboratory of Virology, Frontier Science Center for Immunology and Metabolism, College of Life Sciences, Wuhan University, Wuhan 430072, China; Y18702772142@163.com

**Keywords:** age-related macular degeneration, complement C1S, adrenomedullin, IER5L, immune cell infiltration

## Abstract

Age-related macular degeneration (AMD) is a progressive neurodegenerative disease of the central retina, with no suitable biomarkers for early diagnosis and treatment. This study aimed to find potential diagnostic biomarker candidates for AMD and investigate their immune-related roles in this pathology. Weight gene correlation analysis was first performed based on data from the Gene Expression Omnibus database and 20 hub genes were identified. The functional enrichment analyses showed that the innate immune response, inflammatory response, and complement activation were key pathways associated with AMD. Complement C1s (C1S), adrenomedullin (ADM), and immediate early response 5 like (IER5L) were identified as the crucial genes with favorable diagnostic values for AMD by using LASSO analysis and multiple logistic regression. Furthermore, a 3-gene model was constructed and proved to be of good diagnostic and predictive performance for AMD (AUC = 0.785, 0.840, and 0.810 in training, test, and validation set, respectively). Finally, CIBERSORT was used to evaluate the infiltration of immune cells in AMD tissues. The results showed that the NK cells, CD4 memory T cell activation, and macrophage polarization may be involved in the AMD process. C1S, ADM, and IER5L were correlated with the infiltration of the above immune cells. In conclusion, our study suggests that C1S, ADM, and IER5L are promising diagnostic biomarker candidates for AMD and may regulate the infiltration of immune cells in the occurrence and progression of AMD.

## 1. Introduction

Age-related macular degeneration (AMD) is a progressive retinal disease and a leading cause of irreversible vision loss in older adults worldwide [1]. With aging populations in many countries, the prevalence of AMD has risen markedly in recent years [2]. The affected individuals globally reached around 200 million by 2020 and are expected to increase to nearly 300 million by 2040, thus posing a major public health problem with substantial socioeconomic implications [3]. According to the severity of fundus lesions, AMD is classified into early, intermediate, and late stages, including geographic atrophy (GA; or “late dry AMD”), choroidal neovascularization (CNV; or “wet AMD”), or both [4]. Because the early diagnosis is still a challenge, the disease progress, for most patients, results in a poor prognosis [5]. Currently, the mechanisms of AMD pathogenesis are poorly defined, making early detection and accurate treatment more difficult [6]. Therefore, it is urgent to explore novel diagnostic biomarkers to further develop therapeutic approaches for AMD patients.

Accumulation of diverse immune cells in the subretinal space is a hallmark feature of the development of AMD [7]. In recent years, more and more studies have indicated that immune cell infiltration may play a critical role in the occurrence and progression of AMD [8]. For example, an increasing population of T cells may lead to the recruitment of peripheral monocytes, trigger the secretion of inflammatory cytokines and complement factors, and ultimately exacerbate the AMD process [9,10]. Niazi et al. showed that the elevated neutrophil-to-lymphocyte ratio may have stronger relevance to the neovascular subtype of AMD [11]. The detailed landscape of immune infiltration remains unclear. Therefore, understanding the profile of immune cell infiltration is important not only for clarifying the molecular mechanism underlying AMD but also for developing new immunotherapeutic targets. CIBERSORT is a novel biology tool that uses gene expression data to determine the component of infiltrating immune cells in samples [12]. It has been widely used in immune cell infiltration analysis in various diseases such as systemic lupus erythematosus [13], osteoarthritis [14], and various cancers [15,16]. However, no studies have so far used CIBERSORT to analyze immune cell infiltration in AMD.

In the present study, we first performed the weighted gene co-expression network analysis (WGCNA) and functional enrichment analyses to identify the hub genes and pathways in AMD based on data from the Gene Expression Omnibus (GEO) database. Subsequently, we identified three crucial genes with diagnostic value for AMD by the least absolute shrinkage and selection operator (LASSO) analysis and logistic regression method and then constructed a diagnostic prediction model for AMD. Furthermore, we used CIBERSORT for the first time to analyze the profile of immune cell infiltration in AMD. The correlation between crucial genes with infiltrating immune cells was analyzed to better understand the molecular immune mechanism during the development of AMD.

## 2. Materials and Methods

### 2.1. Data Processing

The microarray data on gene expression profiles related to AMD were downloaded from the National Center of Biotechnology Information (NCBI) GEO database (https://www.ncbi.nlm.nih.gov/geo/; accessed on 5 March 2021). The dataset GSE29801, containing 63 retinal tissues from AMD patients and 55 normal retinal tissues from healthy persons, was used to identify crucial genes for AMD. The dataset GSE50195, containing 9 retinal tissues from AMD patients and 13 normal retinal tissues from healthy persons, was used as the validation set to verify the diagnostic performance of the AMD prediction model.

The data mining techniques and statistical analysis of this study were based on Bioconductor Packages (http://www.bioconductor.org/; accessed on 5 March 2021) of R software (version 3.6.3; https://www.r-project.org/; accessed on 5 March 2021). The gene probes in raw data of the transcriptome microarray were turned into readable gene symbols according to the platform’s annotation information. The probe that did not match the gene symbol was removed and the average expression values were calculated and adopted if multiple probes correspond to one given gene. The background adjustment was performed using the normalizeBetweenArrays function in the limma package and the justRMA function in the Affy package of R to normalize the gene expression matrix in the datasets GSE29801 and GSE50195, respectively. Then, the quantile correction was performed under the R environment to keep the maximal amount of gene profile information with the lowest possible noise.

### 2.2. Weight Gene Correlation Network Analysis

Genes with the top 25% variance were screened out from GSE29801 to conduct WGCNA [17]. According to the morphological phenotype of the eyes, the patients were classified into normal, pre-AMD, dry AMD, GA, CNV, and GA/CNV groups [18]. The hierarchical clustering analysis was conducted on the 118 samples by the hclust function. The pickSoftThreshold function was used to determine a suitable soft-thresholding power value with the standard of independence degree > 0.8 during module construction. With the best power value of 2 and the minimum module size of 40, the gene modules were identified and each was assigned a unique color label.

The expression profiles of each module were summarized by the module eigengene (ME). The associations of individual genes with each subtype were quantified by gene significance (GS) value. The intramodular connectivity of genes in each module was assessed by the module membership (MM) value, which was defined as the correlation of gene expression profile with the ME value. The modules highly correlated with clinical subtypes of AMD were selected as key modules. Genes with high MM in the key modules were defined as the hub genes.

### 2.3. Functional Enrichment Analysis

To identify the biological functions of the module genes and the key pathways involved in AMD, the gene ontology (GO) enrichment analysis of genes in key modules was implemented in the Database for Annotation, Visualization and Integrated Discovery (DAVID) website (version 6.8; https://david.ncifcrf.gov/; accessed on 5 March 2021). The biologic process (BP), cellular component (CC), and molecular function (MF) terms with the adjusted *p*-value < 0.05 were regarded as statistically significant.

Meanwhile, the gene set enrichment analysis (GSEA) of the gene expression profile from the GSE29801 dataset was performed using the gseGO and gseKEGG functions of the clusterProfiler package in R. The significant BP terms of the GO analysis and the KEGG pathways were identified with the standard of the adjusted *p*-value < 0.05.

### 2.4. Identification of Diagnostic Biomarkers and Model Construction

The LASSO algorithm, a method that is suitable for reducing the dimensions of data, was conducted by the glmnet package in R to screen the optimal gene biomarkers from the hub genes. Genes with non-zero regression coefficients were selected. Subsequently, the dataset GSE29801 was randomly divided into a training set and a test set. The multivariable logistic regression analysis was conducted on the train cohorts to identify the diagnostic biomarker candidates. Based on the identified biomarkers, a nomogram model for AMD was constructed utilizing the rms package of R. The performance of the nomogram model was evaluated by the calibration curve. Furthermore, the ROC curve was drawn by the ROCR package in R to compare the diagnostic efficiency of the nomogram model with that of basic demographic characteristics, including age and gender, in the training, test, and validation set.

### 2.5. Immune Infiltration Analysis in AMD

The immune cell infiltration analysis on dataset GSE29801 was conducted by CIBERSORT, and analyses were performed based on samples with *p*-value < 0.05. The profile of the infiltrating immune cells was visualized by the barplot, heatmap, and violin diagram. Unpaired *t*-test (with Welch’s correction if *F* test *p* < 0.05) was used to compare infiltration levels of immune cells between different groups. The corrplot package of R was used to draw a correlation heatmap to visualize the correlation between 22 types of infiltrating immune cells in AMD samples. To further investigate the immune-related role of the identified biomarkers in AMD, the Pearson correlation analysis on the gene expression and the immune cell infiltration was performed by the cor.test function of R and was visualized by the dotplot with the ggplot2 package of R.

## 3. Results

### 3.1. Hub Genes and Modules Associated with AMD

**Figure 1:**
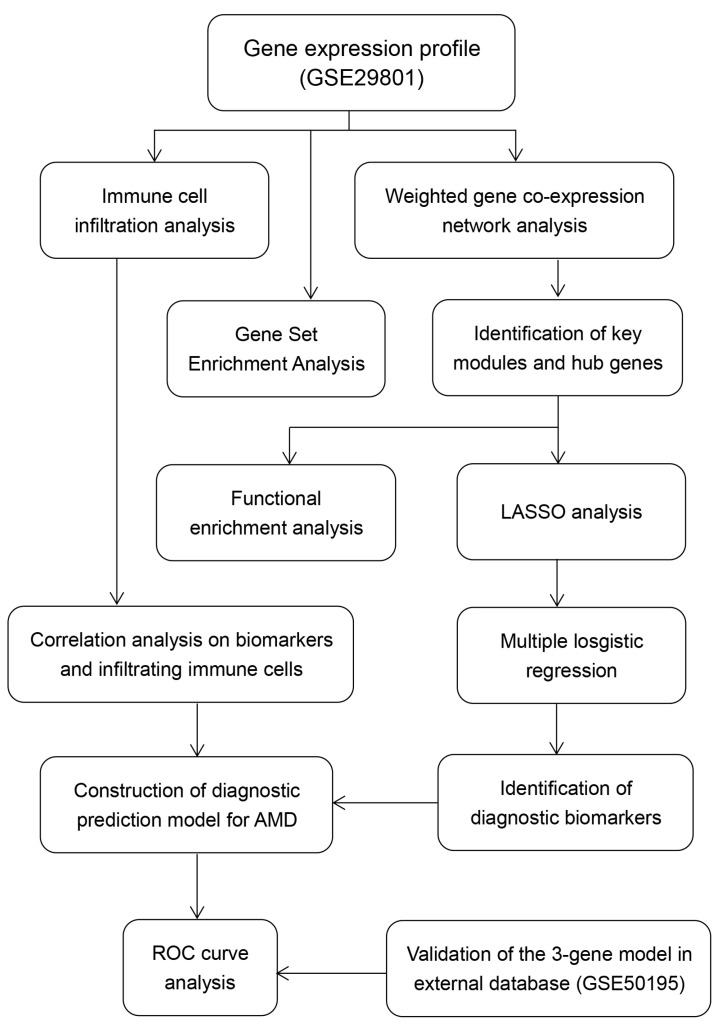
The workflow of the present study.

**Figure 2:**
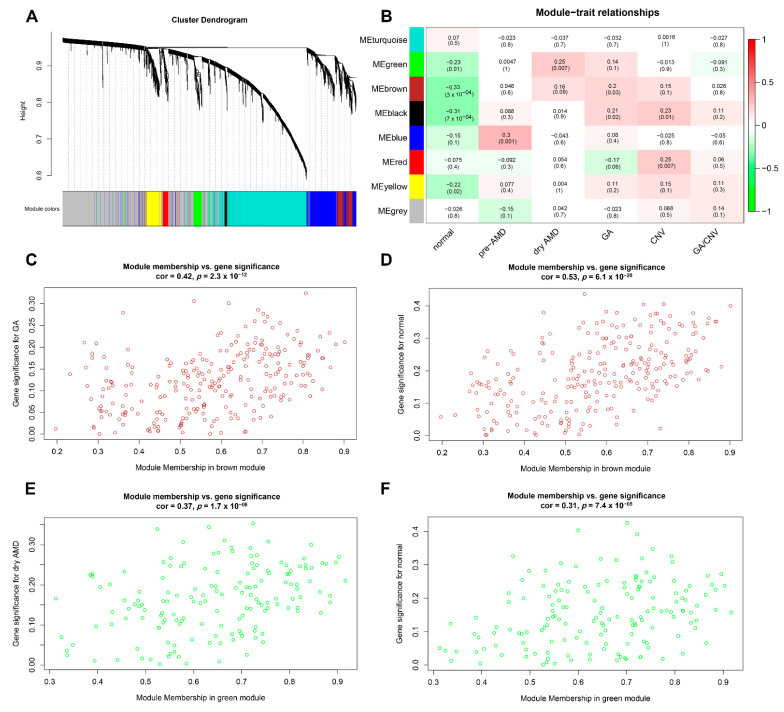
Weighted gene co-expression network analysis. (**A**) Eight modules were identified. (**B**) The correlation between modules and disease subtypes, red indicates positive correlation and green indicates negative correlation. (**C**,**D**) The correlation between module membership in the brown module with gene significance for geographic atrophy (r = 0.42, *p* < 0.001) and normal group (r = 0.53, *p* < 0.001), respectively. (**E**,**F**) The correlation between module membership in the green module with gene significance for dry AMD (r = 0.37, *p* < 0.001) and normal group (r = 0.31, *p* < 0.001), respectively.

Using the workflow shown in Figure 1, a total of eight modules were identified based on 4937 genes from 118 samples (Figure 2A). The number of genes per module is shown in Supplementary Table S1. As shown in Figure 2B, the brown module had a significantly negative correlation with the normal subtype (r = −0.33; *p* < 0.001) and a significantly positive correlation with the GA subtype (r = 0.20; *p* = 0.03); the green module had a significantly negative correlation with the normal subtype (r = −0.23; *p* = 0.01) and a significantly positive correlation with the dry AMD subtype (r = 0.25; *p* = 0.007); the black module had a significantly negative correlation with the normal subtype (r = −0.31; *p* < 0.001) and a significantly positive correlation with the GA (r = 0.21, *p* = 0.02) and CNV subtypes (r = 0.23, *p* = 0.01).

The correlation between GS and MM indicated that the brown and green modules were significantly associated with AMD subtypes (Figure 2C–F). The top 20 genes with high MM in brown and green modules were identified as hub genes (C1S, IFI30, HLAF, CIR, HLAC, CSF1R, CDA12, C1QB, ANXA1, ITGB2, GDF15, EFNA1, ICAM1, CD44, CEBPD, ANGPTL4, ADM, IER5L, MST150, VEGFA; Supplementary Table S2).

### 3.2. Biological Processes and Key Pathways Involved in AMD

**Figure 3:**
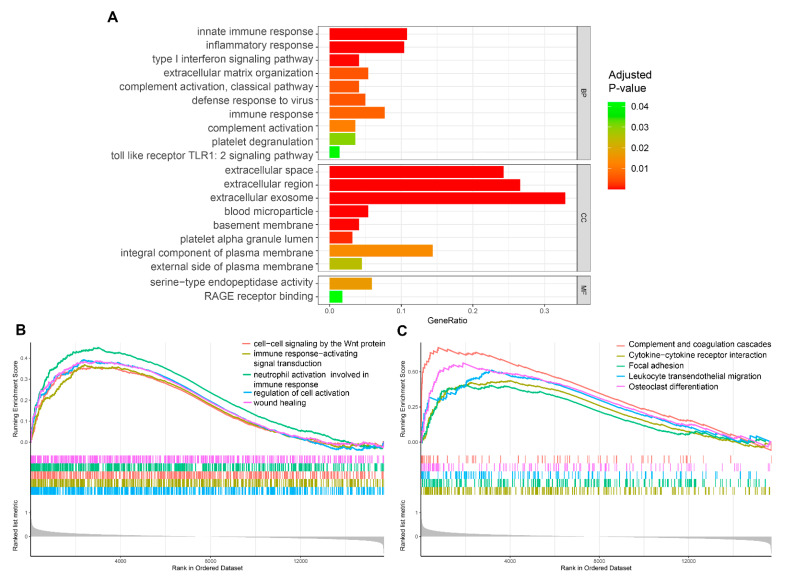
Functional enrichment analysis. (**A**) Biological processes, molecular functions, and cellular components enriched in AMD by gene ontology analysis. (**B**) Biological processes enriched in AMD by gene set enrichment analysis (GSEA). (**C**) KEGG pathways enriched in AMD by GSEA.

The module function enrichment analysis showed that the biological processes were significantly related to innate immune response, inflammatory response, type I interferon signaling pathway, and complement activation; the cellular components were significantly related to extracellular space, region, and exosome; the molecular functions were significantly related to serine-type endopeptidase activity and RAGE receptor binding (adjusted *p*-value < 0.05; Figure 3A).

Meanwhile, the results of the GSEA analysis showed that the biological processes associated with AMD were significantly enriched in cell–cell signaling by the Wnt protein, immune response-activating signal transduction, neutrophil activation involved in immune response, and regulation of cell activation (Figure 3B). The key pathways of AMD were significantly enriched in complement and coagulation cascades, cytokine-cytokine receptor interaction, focal adhesion, and leukocyte transendothelial migration (Figure 3C).

### 3.3. Diagnostic Biomarker Candidates and Prediction Model for AMD

**Figure 4:**
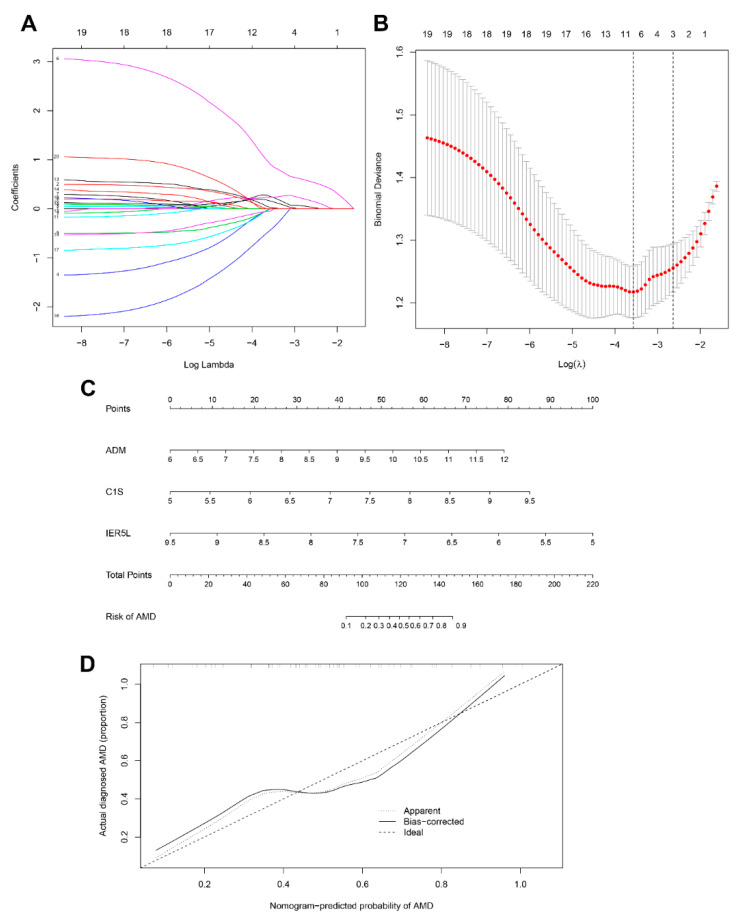
Diagnostic prediction model for AMD. (**A**,**B**) LASSO model. (**C**) Nomogram model for AMD prediction based on C1S, ADM, and IER5L. (**D**) Calibration curve of the 3-gene model.

**Figure 5:**
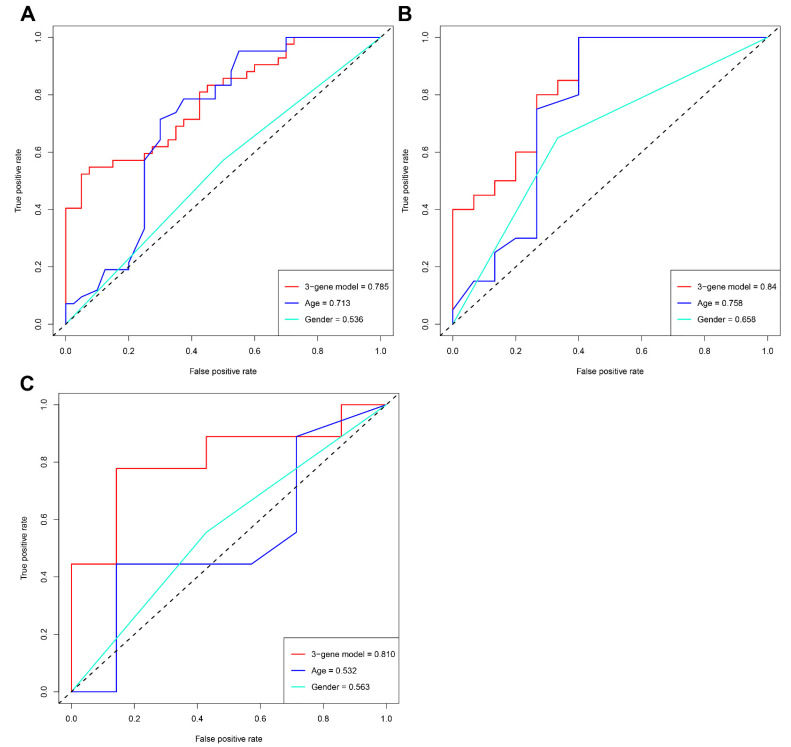
Comparing the differences of our classifier with demographic characteristics. ROC curves of the 3-gene model, age, and gender in the training (**A**), test (**B**), and validation set (**C**).

Using the LASSO method, 20 hub genes were reduced to eight potential predictors with non-zero regression coefficients and the value of lambda.min = 0.03406166 (Figure 4A,B, Supplementary Table S3). According to further multivariable logistic regression analysis, complement C1s (C1S), adrenomedullin (ADM), and immediate early response 5 like (IER5L) were identified as the diagnostic biomarker candidates for AMD [OR (95%CI): 11.302 (2.485, 655.7), *p* = 0.003; OR (95%CI): 3.048 (1.368, 7.790), *p* = 0.011; OR (95%CI): 0.119 (0.293, 0.375), *p* < 0.001, respectively] (Table 1). A nomogram model based on the three genes was established for AMD diagnosis and prediction (Figure 4C). The calibration curve showed excellent agreement between the prediction by nomogram and the actually observed probability of AMD (Figure 4D). The ROC curve analyses indicated that the AUC of the 3-gene-based model was 0.785 in the training cohorts (Figure 5A), 0.840 in the test cohorts (Figure 5B), and 0.810 in the validation cohorts (Figure 5C).

In addition, the clinical benefit of the 3-gene model was compared with that of demographic characteristics, including age and gender. The AUC values of the 3-gene model were greater than both age and gender in the training, test, and validation sets, suggesting that our model had a better diagnostic efficiency for AMD (Figure 5).

### 3.4. Profile of Immune Cell Infiltration in AMD

**Figure 6:**
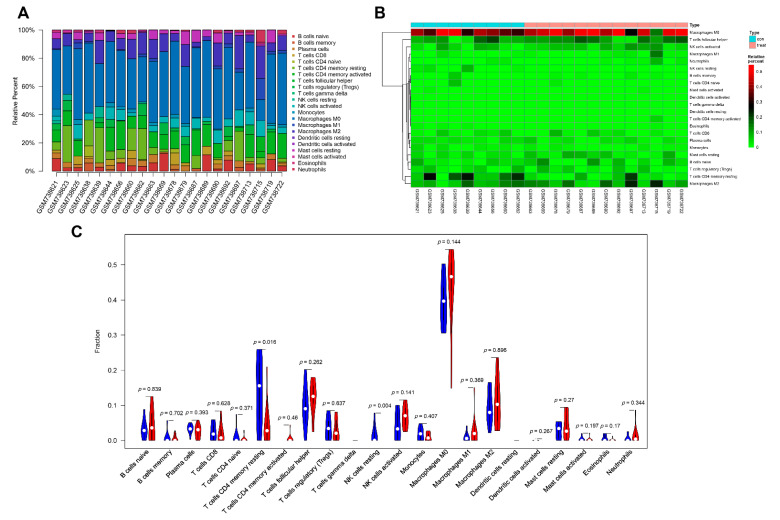
The profile of infiltrating immune cells. (**A**) the proportion of 22 kinds of infiltrating immune cells. (**B**) The heatmaps of the subpopulations of immune cells. (**C**) The violin plot compared the profile of infiltrating immune cells between AMD samples and normal samples; blue represents normal samples and red represents AMD samples.

The immune cell infiltration analysis suggested a significant difference between AMD and normal retinal tissues. The infiltration of 22 kinds of immune cells in these samples is summarized in Figure 6A and the subpopulations of immune cells identified by unsupervised hierarchical clustering are shown in Figure 6B. The violin plot showed that relative to normal control samples, lower proportions of resting NK cells (*p* = 0.004) and resting CD4 memory T cells (*p* = 0.016) were detected in AMD samples (Figure 6C).

**Figure 7:**
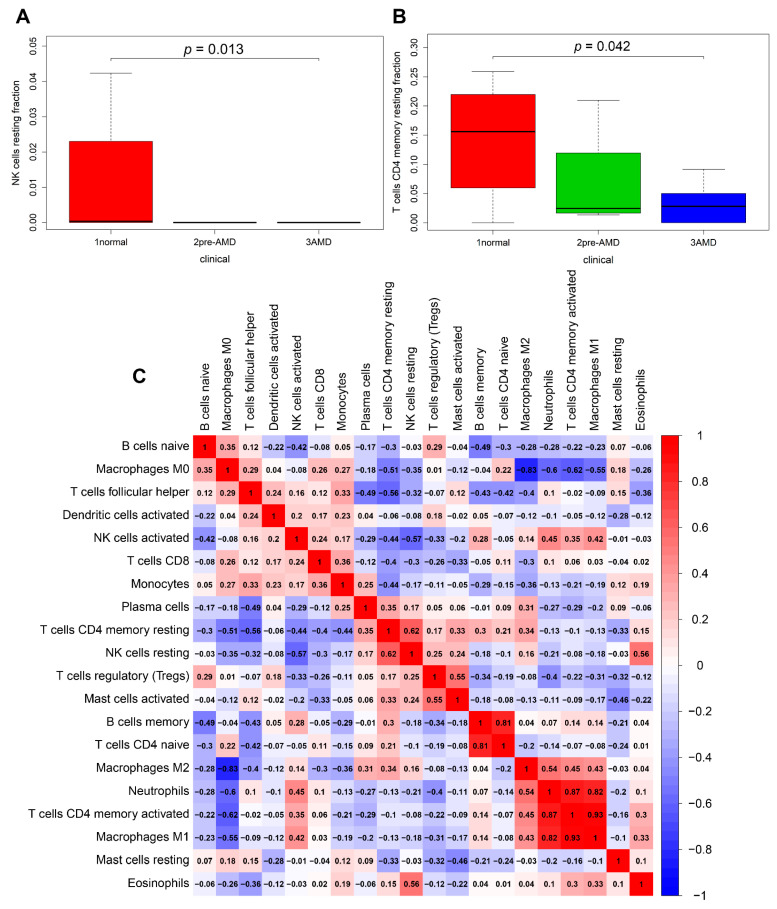
Clinical relevance and correlation analysis of the infiltrating immune cells. The association between the progress of AMD with the resting NK cells (**A**) and resting CD4 memory T cells (**B**). (**C**) Correlation analysis between immune cell types; red indicates positive correlation and blue indicates negative correlation.

The infiltration levels of the resting NK cells and resting CD4 memory T cells were significantly associated with the progress of AMD (*p* = 0.013, *p* = 0.042, respectively) (Figure 7A,B). The correlation analysis between immune cell types revealed that the infiltration level of the activated CD4 memory T cells was negatively correlated with that of macrophages M0 (r = −0.82) and positively correlated with that of macrophages M1 (r = 0.93); the infiltration level of macrophages M0 was negatively correlated with that of macrophages M1 (r = −0.55) and macrophages M2 (r = −0.83) (Figure 7C).

Together, these results indicated that aberrant immune infiltration and its heterogeneity in AMD as a tightly regulated process might have important clinical meanings.

### 3.5. Correlation of Biomarkers with Infiltrating Immune Cells

**Figure 8:**
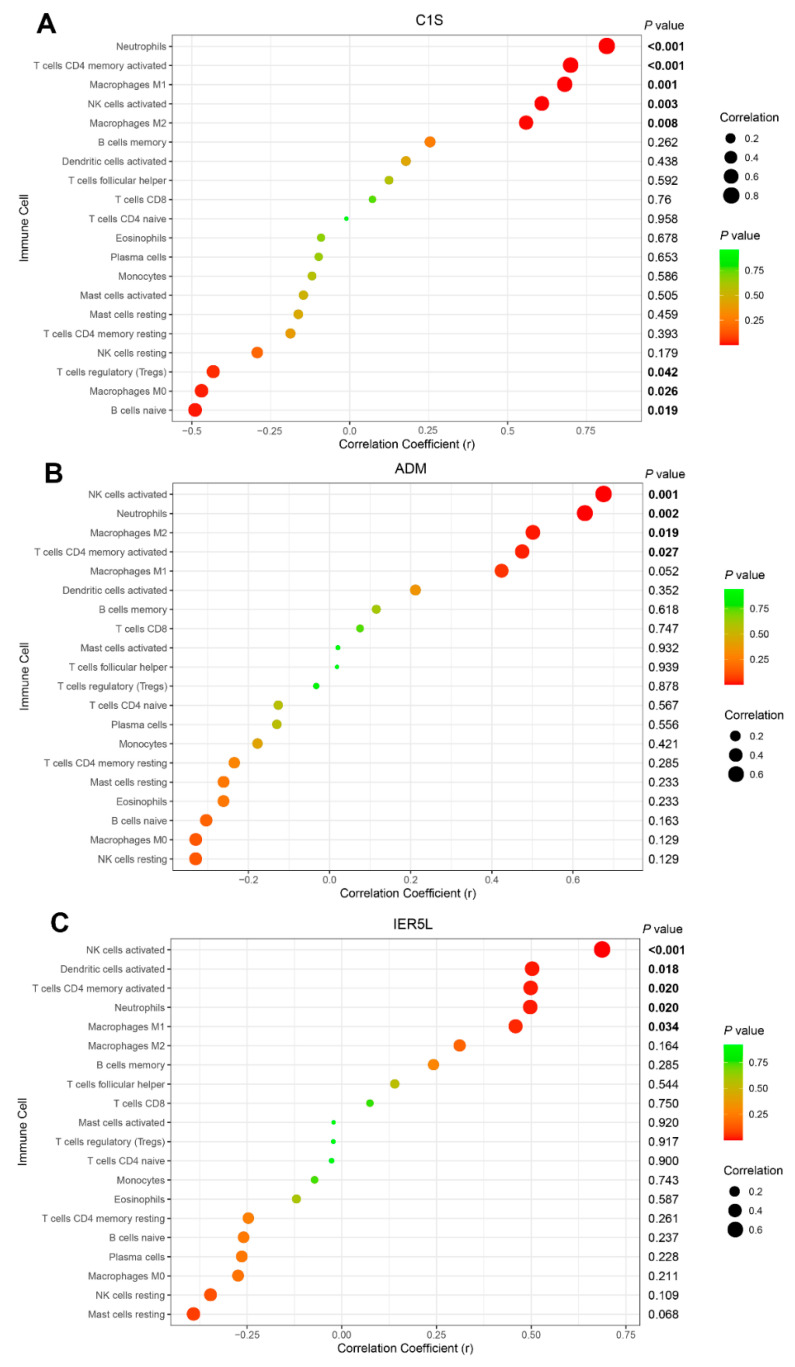
The correlation analysis between the infiltration levels of immune cells with C1S (**A**), ADM (**B**), and IER5L (**C**), respectively.

Correlation analysis showed that C1S was positively correlated with the infiltration levels of neutrophils (r = 0.807; *p* < 0.001), activated CD4 memory T cells (r = 0.693; *p* < 0.001), macrophages M1 (r = 0.674; *p* = 0.001), activated NK cells (r = 0.602; *p* = 0.003), and macrophages M2 (r = 0.553; *p* = 0.008) and negatively correlated with naïve B cells (r = −0.495; *p* = 0.019), macrophages M0 (r = −0.475; *p* = 0.026), and regulatory T cells (r = −0.437; *p* = 0.042) (Figure 8A); ADM was positively correlated with the infiltration levels of activated NK cells (r = 0.670; *p* = 0.001), neutrophils (r = 0.624; *p* = 0.002), macrophages M2 (r = 0.500; *p* = 0.019), and activated CD4 memory T cells (r = 0.470; *p* = 0.027) (Figure 8B); IER5L was positively correlated with activated NK cells (r = 0.682; *p* < 0.001), activated dendritic cells (r = 0.498; *p* = 0.018), activated CD4 memory T cells (r = 0.494; *p* = 0.020), neutrophils (r = 0.493; *p* = 0.020), and macrophages M1 (r = 0.454; *p* = 0.034) (Figure 8C).

## 4. Discussion

AMD is a chronic retinal degenerative disease affecting millions of people worldwide and far from being fully understood and treated [19]. Novel molecular biomarkers for early diagnosis and effective treatment are urgently required. An increasing number of studies suggest that immune dysregulation is a critical process in the onset and progression of AMD [20]. However, the underlying mechanisms are poorly defined. The analysis of candidate biomarkers and immune cell infiltration is of clinical benefit to the diagnostic and therapeutic strategies for AMD. In this study, we sought to identify the promising diagnostic biomarkers for AMD and further explore their immune-related role in AMD via bioinformatics methods.

Based on the AMD expression profile dataset from the GEO database, we applied the WGCNA analysis and found the brown and green modules to be significantly associated with clinical progression. The module function enrichment analysis indicated the involvement of innate immune response, inflammatory response, type I interferon signaling pathway, and complement activation in AMD. The GSEA also identified the pathways related to Wnt signaling, immune response, complement activation, etc. The above results suggested that immune dysregulation plays an important role in AMD. According to previous experimental studies, the Wnt signaling was found aberrantly increased in wet AMD, contributing to pathological angiogenesis [21,22]. The complement system was also demonstrated to have a causative role in AMD development and had been introduced into emerging clinical trials as a potential therapeutic target [23,24]. These findings are consistent with those from our study, suggesting that our analysis results are accurate and may provide important referential merit for clinical applications of AMD.

Molecular marker-based prediction models of diseases with insidious onsets have considerable potential to help early diagnosis, showing a promising prospect in clinical use [25,26,27]. In this study, we identified three candidate diagnostic biomarkers—C1S, ADM, and IER5L—for AMD and then introduced them into the nomogram model for AMD diagnosis and prediction. The priority and stability of the 3-gene model proved to be good by both internal and external validation. Besides, we also revealed the superiority of our 3-gene model in predicting AMD diagnosis compared to age and gender. Previously, aging was considered as the strongest demographic marker for AMD, and gender (females are affected more) as an additional risk factor [28]. The current work constructed a more robust classifier and uncovered more critical information that would benefit the diagnosis and treatment of AMD patients.

C1S is a serine protease and a major constituent of the complement subcomponent C1 [29]. The inhibition of C1S can block the complement cascade at an early stage [30]. Given the crucial role of C1S in the classical complement pathway, we believe that C1S is likely to be involved in regulating the pathological process of AMD and is expected to become a new target for AMD diagnosis and treatment. ADM is a 52-amino-acid multifunctional peptide and belongs to the calcitonin gene-related peptide superfamily of vasoactive peptide hormones. It intervenes in neuronal dysfunction through mechanisms such as immune and inflammatory response, apoptosis, or calcium dyshomeostasis [31]. A previous study reported that ADM can function as a key angiogenic mediator of retinal vascularization and contribute to retinochoroidal disease [32]. In this study, we found ADM expression significantly upregulated in the AMD retina compared with normal controls and that it had the potential to serve as the diagnostic biomarker for AMD. We speculate that ADM plays an important role in AMD development and may have the clinical utility of the targeted therapies. IER5 is one of the growth factor-inducible genes and is reported to be associated with the poor prognosis of cancer patients [33]. IER5L is named as an IER5-like gene, and its cellular roles have not been elucidated [34]. The results of our study showed that IER5L is significantly lower expressed in the AMD retina than in normal controls and exhibits a good diagnostic value for AMD. However, numerous experimental and clinical studies are still needed to verify the expression pattern and diagnostic value of IER5L.

A comprehensive evaluation of AMD immune cell infiltration was additionally conducted in this study. The results showed that a decreased proportion of resting NK cells and resting CD4 memory T cells occurs in the process of AMD. Besides, the activated CD4 memory T cell infiltration is negatively related to the infiltration of macrophages M0 and positively related to the infiltration of macrophages M1. According to a previous study, macrophage M1 polarization can induce the proliferation and migration of human choroidal vascular endothelial cells and therefore induce choroidal neovascularization [35]. Wu et al. showed that patients with the wet-type AMD presented significantly higher levels of CD4 T cells than non-AMD controls [36]. Our results are consistent with the above findings, which further suggest that the CD4 memory T cell activation and macrophage M1 polarization play important roles in AMD and should be the highlight of further studies. However, no research has been conducted on the role of NK cells in AMD, and further experimental data are required. Our results also suggested that C1S, ADM, and IER5L raise activated NK cells, neutrophils, polarized macrophages, and activated CD4 memory T cells to participate in the occurrence and progress of AMD. However, these mechanisms are based on bioinformatics results, and molecular experiments should follow to further validate them.

## 5. Conclusions

In conclusion, the present study found that C1S, ADM, and IER5L are promising diagnostic biomarker candidates for AMD. Based on the three genes, a diagnostic prediction model was constructed and proved to be of good performance. The heterogeneity of infiltrating immune cells was found involved in AMD pathogenesis. The regulation of infiltrating immune cells by C1S, ADM, and IER5L may play an important role in AMD development.

## Figures and Tables

**Table 1:** The results of multiple logistic regression analysis.

Variable	Multiple Logistic Regression		
	Regression Coefficient	Odds Ratio (95% CI)	*p*-Value
(Intercept)	1.142	3.134 (3.930 × 10^−7^, 2.449 × 10^7^)	0.887
ADM	1.115	3.048 (1.368, 7.790)	0.011 *
C1S	2.425	11.302 (2.485, 655.7)	0.003 **
CSF1R	−1.707	0.181 (0.020, 1.347)	0.105
HLAC	−0.663	0.516 (0.049, 5.079)	0.571
HLAF	1.115	3.050 (0.309, 3507)	0.349
IER5L	−2.128	0.119 (0.293, 0.375)	<0.001 ***
ITGB2	−0.331	0.718 (0.222, 2.246)	0.570
MST150	0.305	1.357 (0.557, 3.355)	0.499

ADM, adrenomedullin; C1S, complement C1s; CSF1R, colony stimulating factor 1 receptor; HLAC, major histocompatibility complex, class I, C; HLAF, major histocompatibility complex, class I, F; IER5L, immediate early response 5 like; ITGB2, integrin subunit beta 2; MST150, small integral membrane protein 3; * *p* < 0.05; ** *p* < 0.01; *** *p* < 0.001.

## Data Availability

All data, models, or codes generated or used during the study are available from the corresponding author on request.

## Supplementary Materials

The following are available online at https://www.mdpi.com/article/10.3390/diagnostics11061079/s1, Table S1: Number of genes in different modules, Table S2: The MM value of the top 20 genes in brown and green modules, Table S3: The eight genes identified by LASSO analysis.
