# Supplementary material for: Identification of Diagnostic Biomarkers and Their Correlation with Immune Infiltration in Age-Related Macular Degeneration
Source: Diagnostics (Basel). 2021 Jun 12;11(6):1079. doi: 10.3390/diagnostics11061079 (PMC8231534; doi:10.3390/diagnostics11061079)
Supplement: Supplementary file 1 [file diagnostics-11-01079-s001.zip › Supplementary Table S1-S3.pdf]

Table.S1 Number of genes in different modules.

module	number
grey	1972
turquoise	1466
blue	686
brown	256
yellow	242
green	158
red	90
black	67
total	4937

Table.S2 The MM value of the top 20 genes in brown and green modules.

genes	MM value	description
Brown module		
C1S	0.901466536	complement C1s
IFI30	0.879731229	IFI30 lysosomal thiol reductase
HLAF	0.866933111	major histocompatibility complex, class I, F
C1R	0.864557406	complement C1r
HLAC	0.849045857	major histocompatibility complex, class I, C
CSF1R	0.846843588	colony stimulating factor 1 receptor
CDA12	0.845843318	major histocompatibility complex, class I, F
C1QB	0.842099467	complement C1q B chain
ANXA1	0.835827291	annexin A1
ITGB2	0.824006771	integrin subunit beta 2
Green module		
GDF15	0.916652932	growth differentiation factor 15
EFNA1	0.903036343	ephrin A1
ICAM1	0.89619404	intercellular adhesion molecule 1
CD44	0.884552481	CD44 molecule
CEBPD	0.884421184	CCAAT enhancer binding protein delta
ANGPTL4	0.876676809	angiopoietin like 4
ADM	0.868352637	adrenomedullin
IER5L	0.865008795	immediate early response 5 like
MST150	0.863752968	small integral membrane protein 3
VEGFA	0.845785191	vascular endothelial growth factor A

MM, module membership.

Table.S3 The eight genes identified by LASSO analysis.

Variable	Description	Regression coefficient
(Intercept)		-8.489197894
ADM	adrenomedullin	0.249900662
C1S	complement C1s	0.903838776
CSF1R	colony stimulating factor 1 receptor	-0.002686757
HLAC	major histocompatibility complex, class I, C	0.237246169
HLAF	major histocompatibility complex, class I, F	0.024143361
IER5L	immediate early response 5 like	-0.435100807
ITGB2	integrin subunit beta 2	-0.046894269
MST150	small integral membrane protein 3	0.156973431

LASSO, least absolute shrinkage and selection operator.
